# Individualized Frequency‐Dependent Stimulation to the Anterior Nucleus of the Thalamus May Improve Antiepileptic Effects in Patients With Focal Epilepsy

**DOI:** 10.1111/cns.70611

**Published:** 2025-09-10

**Authors:** Ying Gao, Hao Yan, Xueyuan Wang, Guiliang Hao, Liang Qiao, Wei Shu, Duanyu Ni, Guoguang Zhao, Liankun Ren, Tao Yu

**Affiliations:** ^1^ Department of Functional Neurosurgery, Beijing Institute of Functional Neurosurgery, Xuanwu Hospital Capital Medical University Beijing China; ^2^ Department of Neurosurgery, Xuanwu Hospital Capital Medical University Beijing China; ^3^ Department of Neurology, Xuanwu Hospital Capital Medical University Beijing China

**Keywords:** ANT stimulation, epilepsy, Granger causal flow, power spectral density, stereoelectroencephalography

## Abstract

**Aim:**

A total of 30% of individuals with epilepsy are resistant to drug treatment. Deep brain stimulation (DBS) of the anterior nucleus of the thalamus (ANT) shows promise for treating drug‐resistant epilepsy (DRE), but further research is needed to optimize DBS parameters, including stimulation frequency. This study aimed to reveal the optimal frequency for ANT‐DBS by testing the real‐time effects of various stimulation frequencies on the ANT among patients undergoing stereoelectroencephalography (SEEG) electrode implantation.

**Methods:**

Eleven patients (8 males; mean age, 24.2 years; mean epilepsy duration, 13.5 years) were enrolled. Postoperative electrode reconstruction identified ANT contacts for bipolar stimulation between 10 Hz and 100 Hz at 10‐Hz increments, with a 1‐min interval between stimuli. The effects were analyzed on the basis of spike count, power spectral density, and causal flow.

**Results:**

Our study revealed that ANT stimulation suppresses seizure focus activity and modulates brain network dynamics, with the effects varying by individual characteristics and frequency. Stimulation at 10–70 Hz reduced spikes (> 50%) and PSD (*p* < 0.05) in the epileptogenic region while also influencing network connectivity between the epileptogenic zone and adjacent cortical regions, including the frontal, temporal, and insular lobes, as well as the overall brain network.

**Conclusions:**

Instead of using a fixed high frequency for ANT‐DBS, the optimal frequency should be selected for each patient. Patients who do not respond well to high‐frequency ANT‐DBS can use a lower frequency, such as 10–70 Hz.

## Graphical abstract

ANT stimulation exerts a significant inhibitory effect on epileptogenic regions and modulates the large‐scale brain network. These effects exhibit notable interindividual variability and are frequency‐dependent. For epilepsy patients who show a suboptimal response to high‐frequency ANT‐DBS, the use of lower frequencies (10–70 Hz) may improve therapeutic outcomes.

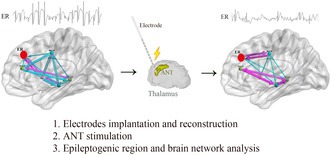

## 1 Introduction

Epilepsy is one of the most common neurological disorders and affects people of all ages [1, 2]. Up to 30% of individuals with epilepsy do not respond to antiseizure drug treatment [3, 4, 5]. Resection surgery provides a greater chance for seizure remission for individuals with drug‐resistant epilepsy (DRE) [6, 7, 8]. However, many patients with DRE do not have a single identifiable seizure focus that is suitable for resection, even if they have focal seizures [9]. Neuromodulation, such as DBS, especially to the anterior nucleus of the thalamus (ANT), is a promising alternative [10, 11, 12, 13]. Although previous clinical studies have confirmed the efficacy of ANT‐DBS for DRE, researchers continue to investigate methods to improve its efficiency [14, 15, 16, 17]. The typical parameters used for ANT‐DBS (pulse width from 90 to 450 μs, voltage from 1 to 5 V, frequency from 130 to 150 Hz, 1 min on/5 min off) might be too rigid for complex brain networks in epilepsy patients [18, 19, 20]. The use of high‐stimulation frequencies, such as in the treatment of Parkinson's disease, may not be suitable for all epilepsy patients, and the current mode may be limited in terms of its potential to exert an antiepileptic effect [21, 22]. We aimed to ascertain the intrinsic effects of DBS on brain networks in epilepsy patients to optimize the DBS regimen. Seizures may be more effectively controlled with individualized stimulation frequencies. This study focused on the role of frequency variation in modulating the epileptic brain network, with the aim of identifying an individualized optimal frequency for ANT‐DBS in each patient.

## 2 Methods

### 2.1 Sex as an Experimental Variable

Our study examined males and females, and similar findings were reported for both sexes; therefore, sex was not considered a biological variable in this study.

### 2.2 Patient Selection

All participants were referred to the Beijing Institute of Functional Neurosurgery, Xuanwu Hospital, for consultation between January 2019 and December 2023. Each patient who was diagnosed with DRE according to the ILAE Classification of Epilepsies underwent a multidisciplinary presurgical evaluation [23, 24]. The inclusion criteria were as follows: (1) patients who required stereoelectroencephalography (SEEG) to identify the epileptogenic region after evaluation; (2) patients with no contraindications to electrode implantation surgery; (3) patients whose SEEG implantation plan included the passage of electrodes through the opercula–insula to the insula; (4) patients with an active electrode in contact with the ANT; and (5) patients who were able to communicate and therefore cooperate with direct electrical stimulation procedures.

All patients provided informed consent after admission. This study was approved by the Medical Ethics Committee of Xuanwu Hospital, Capital Medical University [LYS2018041].

### 2.3 Presurgical Preparation

High‐resolution 3.0 T MR scans, including spin–echo T1‐weighted, T2‐weighted, fluid‐attenuated inversion recovery (FLAIR) sequences, as well as 3‐dimensional anatomic T1‐weighted axial, sagittal, and coronal sequences covering the whole‐brain volume with 0.8‐ or 1‐mm‐thick sections, were obtained for all patients. Additionally, magnetoencephalography (MEG) and positron emission tomography‐computed tomography (PET–CT) were performed to identify epileptogenic regions in all patients [25]. Long‐term scalp video electroencephalography (vEEG) monitoring was routinely performed to record at least three habitual seizures.

### 2.4 SEEG Electrode Implantation

Patients underwent SEEG electrode implantation, with each electrode containing 8–16 contacts depending on its length (contact length: 2 mm, contact spacing: 1.5 mm). An SEEG electrode targeting the opercula‐insular cortex or posterior‐inferior frontal lobe was carefully adjusted so that its tips could enter the ANT.

### 2.5 Electrode Reconstruction and vEEG Monitoring

Preoperative high‐resolution MR images were compared with postoperative thin‐slice CT images via the open‐source software SPM12 [26], and FreeSurfer, and 3D Slicer were used to reconstruct electrode contacts [27]. The intracranial EEG was sampled at 1024 Hz. Video‐EEG monitoring lasted between 3 and 14 days, during which at least three habitual seizures were recorded for each patient. The epileptogenic region was visually identified by a specialized team.

### 2.6 Electrical Stimulation of the ANT

**FIGURE 1:**
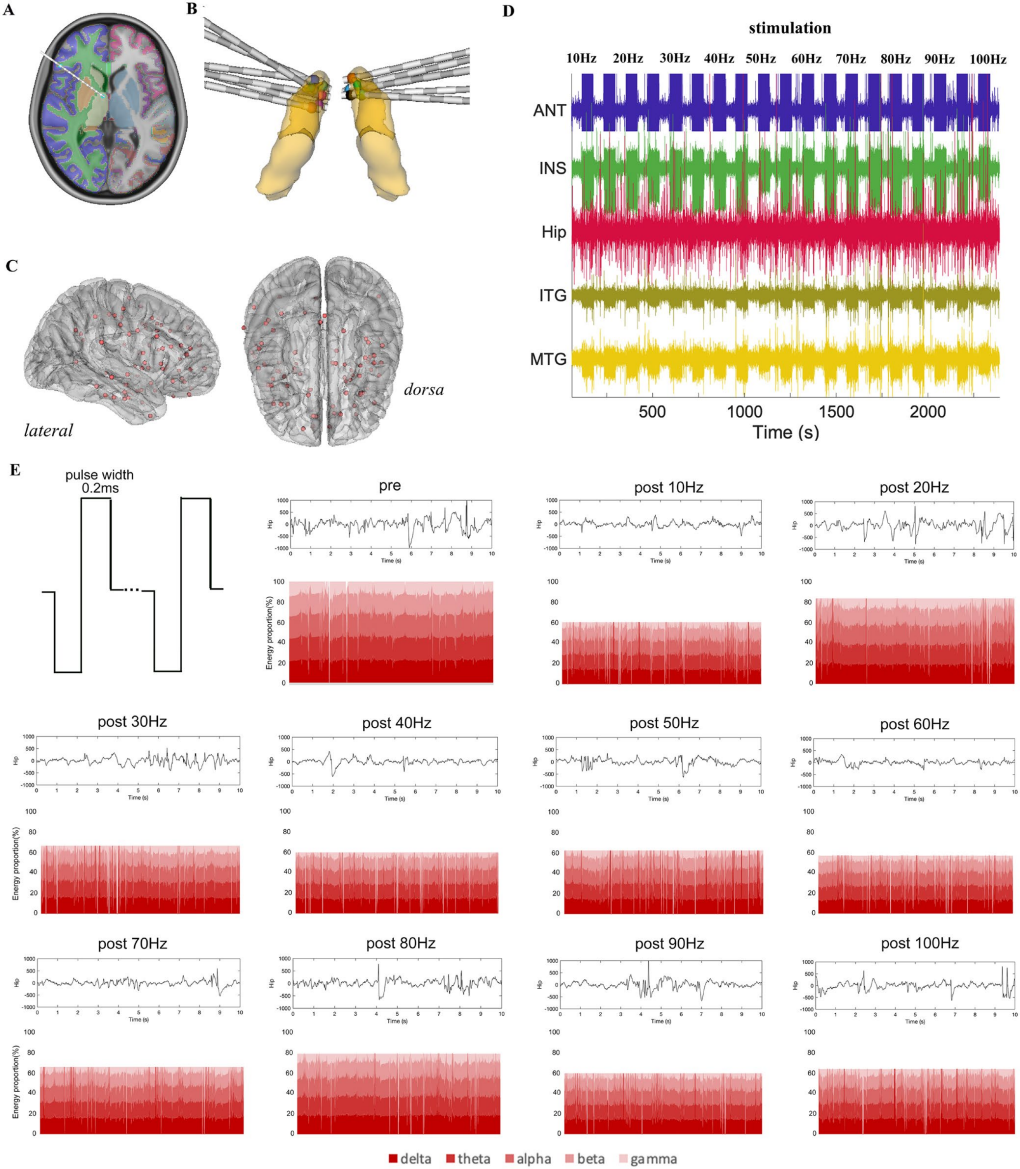
Methods used in the study. (A) Diagram illustrating the electrode trajectory targeting the anterior thalamic nucleus (ANT). (B) This diagram shows the electrode trajectories for all patients in the study, with the ANT highlighted in yellow. (C) This diagram presents the 3D brain surface projection of the regions of interest (ROIs) for all patients in the study. (D) The EEG data from the stimulation process of Patient 2 are shown in this diagram. (E) This diagram depicts the biphasic square‐wave stimulation pattern with a pulse width of 200 μs used in the study, along with changes in the number of spikes and the PSD within the epileptogenic region (hippocampus) for Patient 2 before and after stimulation at various frequencies.

A pair of contacts within the ANT was selected for bipolar stimulation. Each patient underwent testing with an initial current threshold ranging from 0.1 to 1 mA, increasing in increments of 0.5 mA until a clinical response was observed or a maximum current of 6 mA was reached. The highest current at which the patient did not respond to the stimulus was designated the stimulation current. Stimulation commenced at 10 Hz, increasing up to 100 Hz in increments of 10 Hz, with each interval lasting 60 s, followed by a 60‐s rest. SEEG was recorded from all nonstimulated contacts throughout the testing process. The experimental methods used in this study are shown in Figure 1.

### 2.7 Data Processing

First, the regions of interest (ROIs) were identified by selecting 4–8 contacts in each patient's frontal, temporal, and insular gray matter. The criteria for selection were as follows. (1) Extensive fibrous connections between the ANT and the anatomical frontal, medial temporal, and insula lobes indicated ROIs, as described in previous studies. (2) Given that each patient has many implanted electrode contacts, many of which are located within the white matter, the signals recorded from these regions predominantly reflect volume‐conducted potentials from distant cortical generators rather than local neuronal activity. Compared with recordings from gray matter, white matter signals lack the spatial resolution necessary to distinguish discrete neuronal populations and are coupled with diminished temporal specificity, which substantially constrains their utility for phase synchronization analysis and dynamic connectivity assessments. Therefore, the intracranial EEG signals recorded from white matter regions have limited value for analyzing brain network connectivity. (3) When an electrode trajectory passes through a cortical structure, the volume conduction effect can lead to synchronous recordings of the activity of vertically aligned pyramidal neuron populations within a cortical column by adjacent contacts. This approach results in significant spatial correlation among signals recorded from several contiguous contacts, with highly similar spectral features and time‐domain waveforms. (4) The ANT electrode can be contained in the cerebral hemisphere to facilitate analyses. Second, SEEG data were collected when the patient was awake and calm, with no seizures occurring within 2 h before and after the stimulus. Ten seconds of SEEG data, free from artifacts, were selected as the prestimulus baseline. For poststimulus data, a 10‐s segment was chosen after the stimulus artifact had dissipated and SEEG signals had stabilized following different stimulation frequencies.

### 2.8 EEG Signal Preprocessing and Data Selection

The raw EEG data were preprocessed using digital filters: a 50‐Hz notch filter was applied to eliminate line noise interference, followed by a 0.5 to 80‐Hz bandpass Butterworth filter (4th‐order) to retain signals within the effective frequency range. All the channels were subsequently visually inspected for artifact identification and screening. Channels exhibiting significant baseline drift (continuous amplitude shifts exceeding ±100 μV) or noise caused by poor electrode contact (e.g., sudden high‐amplitude fluctuations or signal discontinuities) were flagged as invalid and excluded. The remaining channels with no observable artifacts (e.g., electromyographic interference, eye movements, or motion artifacts) and stable signals were retained as valid data for analysis. All preprocessing procedures were implemented using the EEGLAB toolbox (version 2021.1), with cross‐validation performed independently by two researchers to ensure consistency [28].

### 2.9 Spike Counting and PSD Analysis

Contacts from the epileptogenic region were selected for analysis. The number of spikes in the epileptogenic region was counted independently by three experienced electrophysiologists, ensuring that the statistical error within the same SEEG segment remained below 1. We selected the same contact within the epileptogenic region and used MATLAB R2019b to calculate the power spectral density (PSD) for each patient before and after stimulation at various frequencies.

### 2.10 Network Analysis on the Basis of Conditional GC

To analyze cortical–subcortical network connections before and after stimulation across different patients and to reveal individualized neural circuits between regions of interest, we applied conditional Granger causality (GC) based on multiple autoregressive (MVAR) models in I_ROIs^f(t). This approach facilitated high‐precision connectivity analysis of deep nuclei in MATLAB R2019b [29, 30].

### 2.11 Statistical Analyses

In this study, all measurement data are presented as the means ± standard deviations. Data normality was evaluated using the Shapiro–Wilk test in SPSS (version 26.0, IBM), with a *p* value > 0.05 indicating a normal distribution. Parametric tests were performed for normally distributed data, whereas the Wilcoxon rank‐sum test was conducted for nonnormally distributed data. A *p* value < 0.05 indicated statistical significance.

## 3 Results

### 3.1 General Information

Eleven patients were enrolled in this study, including 8 males with a mean age of 24.2 ± 6.0 years (range 15–32 years) and an average epilepsy duration of 13.5 ± 7.8 years (range 5–29 years) (values for continuous variables are expressed as the means ± SDs). All participants presented with focal seizures. Findings from long‐term video EEG monitoring and imaging revealed that epileptogenic foci were predominantly localized to the frontal lobe (*n* = 6), temporal lobe (*n* = 3), and insular lobe (*n* = 2). The average number of implanted electrodes per patient was 8.3 ± 1.2 (range 7–11 electrodes), corresponding to a mean of 122.6 ± 14.4 contacts (range 108–160 contacts), whereas the mean number of selected cortical contacts (excluding the anterior nucleus of the thalamus, ANT) was 5.6 ± 1.2 (range 4–8 contacts). All patients underwent electrical stimulation targeting the ANT, with a mean stimulation current of 3.9 ± 1.3 mA (range 2–6 mA). Notably, SEEG data were unavailable for Patient (Pt.) 9 following stimulation at a frequency of 10 Hz owing to substantial pseudo‐differences. Patient information is comprehensively summarized in Table 1.

**TABLE 1:** General information of the study participants.

Pat	Sex	Age/His (years)	MRI findings	Electrodes/contacts	ANT side	ROIs (*n*, brain regions)	Best frequency (Hz, spikes/PSD)	Current intensity (mA)	Scope of resection	Pathology	Seizure outcome (ILAE)
**1**	M	31/29	L FL	9/128	L	8, **ACC/**IFG/INS/MCC/MFG/AMY/STG/HIP	50	5	L ACC	FCD IIa	ILAE I
**2**	M	15/6	R HIP	8/117	R	4, INS/**HIP/**ITG/MTG	60/70	3	R ATL	FCD I	ILAE I
**3**	F	28/11	N	8/128	L	7, ACC/**SFG/**INS/MFG/MCC/ITG/HIP	100/10	5	L SFG	FCD Ib	ILAE III
**4**	M	31/20	R HIP	8/122	R	4, **HIP/**STG/MFG/INS	60/100	5	R HIP	—	ILAE I
**5**	M	21/10	L HIP	9/126	L	5, INS/AMY/MTG/ITG/**HIP**	70	3	L ATL	—	ILAE I
**6**	M	25/6	N	7/112	L	5, **INS/**MFG/ITG/HIP/MTG	50/10	6	L INS\ATL	FCD Ib	ILAE IV
**7**	F	16/7	N	7/108	L	6, **IFG/**SFG/MFG/INS/HIP/MTG	60/10	5	L IFG	FCD IIa	ILAE I
**8**	F	19/17	N	8/116	R	5, **INS/**MFG/ACC/IFG/HIP	10/70	3	R INS\ATL\OFL	—	ILAE I
**9**	M	26/5	R FL	9/124	R	6, HIP/MTG/ACC/INS/**MFG/**MCC	10	3	R MFG	FCD IIb	ILAE I
**10**	M	22/17	R FL	7/108	R	6, IFG/ACC/INS/**MFG/**MTG/ITG	20/10	2	R MFG	MCD	ILAE I
**11**	M	32/21	N	11/160	L	6, ACC/**MCC/**MFG/INS/AMY/HIP	60	2.5	L MCC	FCD IIa	ILAE I

Abbreviations: ACC, anterior cingulate cortex; AMY, amygdala; ATL, anterior temporal lobe; ER, epileptogenic region; F, female; FCD, focal cortical dysplasia; FL, frontal lobe; Hip, hippocampus; His, history; IFG, inferior frontal gyrus; ILAE, International League Against Epilepsy; INS, insular; ITG, inferior temporal gyrus; L, left; M, male; MCC, medial cingulate cortex; MCD, malformations of cortical development; MFG, middle frontal gyrus; MTG, middle temporal gyrus; N, negative; OFL, orbital frontal lobe; Pat, patient; R, right; SFG, superior frontal gyrus; STG, superior temporal gyrus; y, years.

### 3.2 Stimulus Changes in the Number of Spikes

**FIGURE 2:**
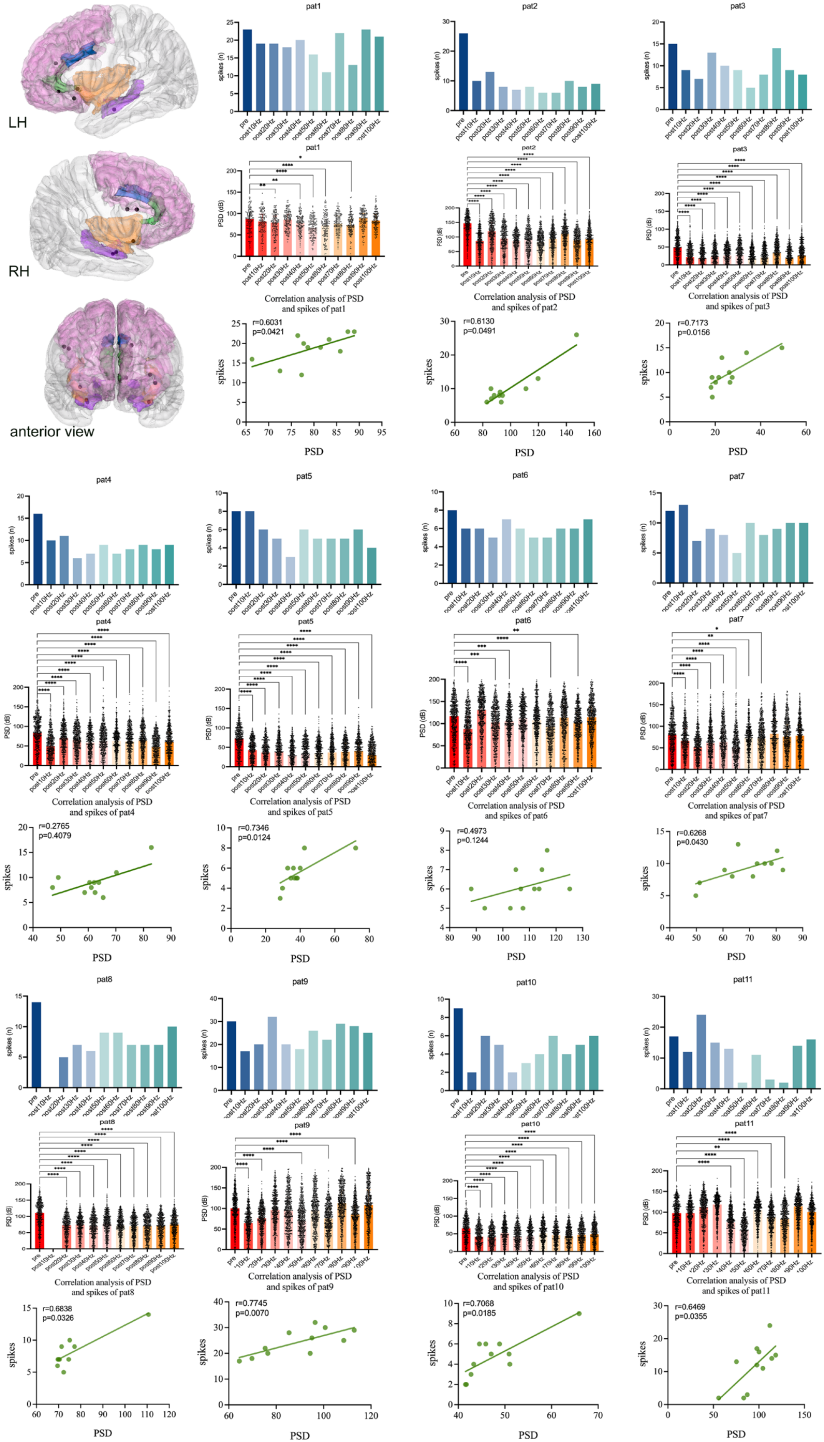
Changes in the number of spikes and the PSD before and after stimulation at each frequency, along with their correlations. The top‐left image displays the location of the epileptogenic regions for all patients in the study, with black dots indicating the electrode contacts within the epileptogenic region, representing the epileptogenic region location. For each patient, three panels are shown: From top to bottom, these panels include changes in the number of spikes within the epileptogenic region before and after stimulation at each frequency, changes in the PSD, and the correlation between the number of spikes and PSD. *p < 0.05; **p < 0.01; ***p < 0.001; ****p < 0.0001. The results have been subjected to the FDR correction.

According to our analysis, the mean number of spikes in the epileptogenic region before stimulation was 16. Following stimulation at various frequencies (10–100 Hz), the average number of spikes was 10, 11, 11, 9, 8, 9, 9, 10, 11, and 11, respectively. Compared with that before stimulation, the number of spikes decreased after each frequency stimulation. Notably, the change in the number of spikes varied across stimulation frequencies (Figure 2), with each patient exhibiting at least one frequency at which the number of spikes decreased by more than 50%.

**FIGURE 3:**
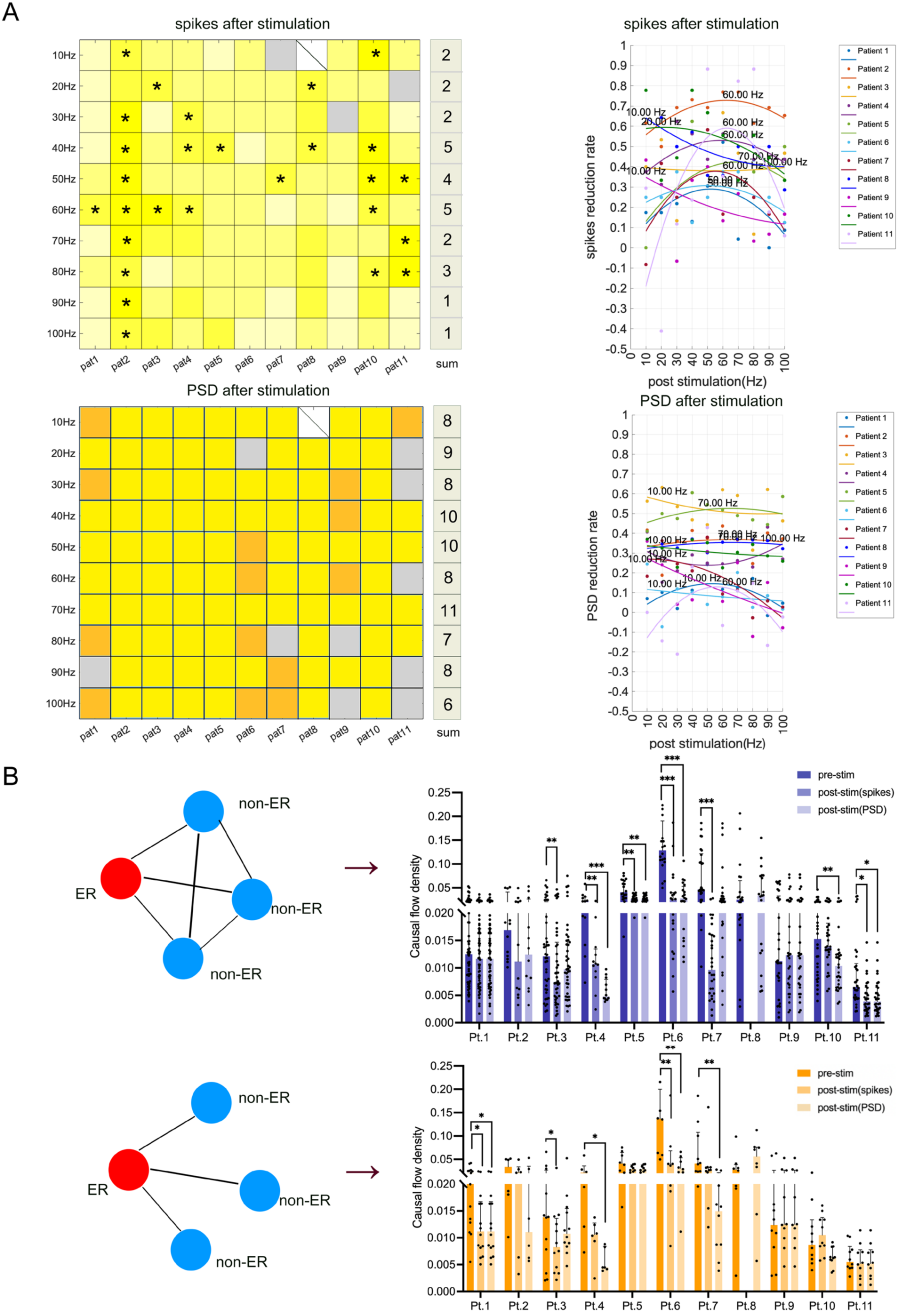
Changes in the number of spikes and PSD within the epileptogenic region and network changes inside and outside the epileptogenic region at the optimal stimulation frequency. (A) The top‐left panel displays the statistical results of the changes in the number of spikes before and after stimulation within the epileptogenic region for all patients in this study. The yellow color gradient indicates the rate of decrease in the number of spikes, from low to high, with a reduction greater than 50% marked by an asterisk ‘*’. The gray areas indicate no reduction in the number of spikes, whereas missing data are represented by ‘\’. The bottom‐left panel shows the statistical results of changes in PSD before and after stimulation within the epileptogenic region for all patients. Yellow represents a decrease in PSD compared with that before stimulation, whereas deep yellow and blue indicate statistically significant reductions. The gray areas indicate no decrease, with missing data marked by ‘\’. The summaries on the right of both panels show the number of patients with a reduction > 50% and statistically significant changes at each frequency. The two right panels display the optimal stimulation frequency for each patient, as predicted through regression analysis using spikes and PSD. (B) This panel shows changes in causal flow density for all patients following stimulation at the optimal frequency. The blue bars represent network changes across all the ROIs selected for each patient, whereas the yellow bars represent changes in the network within the epileptogenic region and between the region and surrounding brain regions. ER, epileptogenic region; non‐ER, non‐epileptogenic region. Asterisks indicate significance levels: *p < 0.05, **p < 0.01, ***p < 0.001. The results have been subjected to the FDR correction.

We observed that while spike reductions greater than 50% were widely distributed across frequencies, they were predominantly concentrated within the 10–80 Hz range. Furthermore, on the basis of individual patient‐specific changes in the number of spikes before and after stimulation, we predicted the optimal stimulation frequency bands via multivariate regression analysis. The most effective stimulation frequencies were primarily within the 10–70 Hz range. The detailed optimal stimulation frequencies for each patient are shown in Figure 3.

### 3.3 Stimulus Changes in the PSD

**FIGURE 4:**
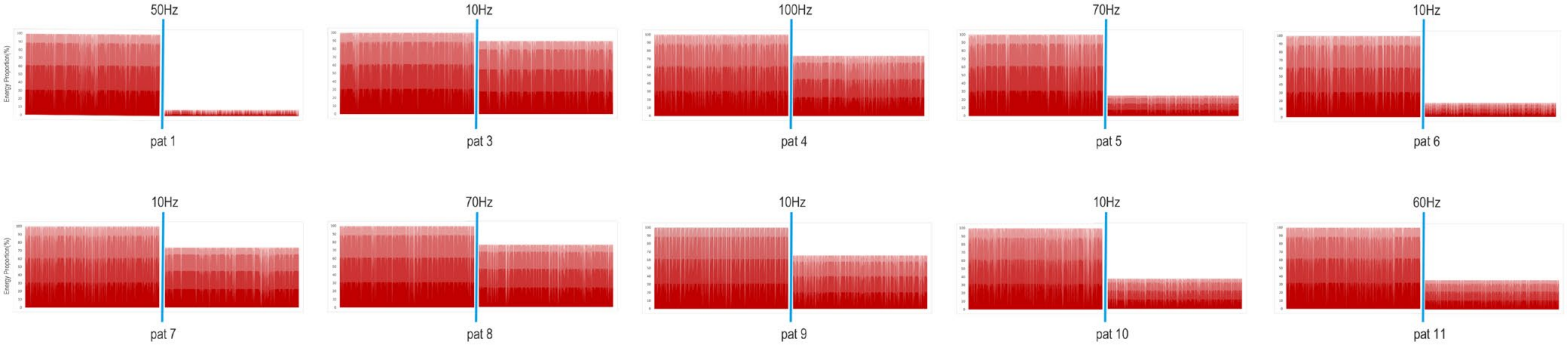
Changes in the PSD before and after stimulation at the optimal frequency. This figure displays the changes in power spectral density (PSD) following stimulation at the optimal frequency, as determined by PSD regression, for all patients except Patient 2. The results for Patient 2 are shown in Figure 1.

We analyzed the PSD for each patient before and after stimulation at various frequencies (10–100 Hz). Despite notable interindividual variability and differing responses to specific frequencies, the overall trend indicated a reduction in PSD after stimulation compared with baseline. For each patient, the PSD significantly decreased at more than half of the stimulation frequencies (Figures 2 and 4).

Notably, the stimulation frequencies associated with significant reductions in PSD were predominantly concentrated within the 10–70 Hz range. Additionally, we predicted the optimal stimulation frequency bands for each patient on the basis of changes in PSD following stimulation at different frequencies via multivariate regression analysis. The results indicated that the most effective stimulation frequencies were predominantly concentrated within the 10–70 Hz range, which aligns with the findings from the spike analysis. Notably, the optimal frequencies identified in two separate regression analyses were consistent across the four patients (Figure 3). Each patient exhibited a decrease in PSD following stimulation at the optimal frequency, as shown in Figure 4. Furthermore, Spearman's correlation analysis revealed a significant association between the number of spikes and PSD in nine patients, as shown in Figure 2.

### 3.4 Stimulus Changes in the Brain Networks

Personalized brain networks were constructed for each patient on the basis of their individually defined regions of interest (ROIs). Using the optimal stimulation frequencies identified through regression analyses of spikes and PSD, we conducted GC analyses on different brain regions for the 11 patients. The results revealed a general reduction in brain network connectivity between the ROIs (frontal lobe, temporal lobe, and insular lobe) following stimulation for all patients except Pt. 8. For several patients, this reduction was statistically significant (optimal frequency on the basis of spikes: Pt. 3: *p* = 0.0036, Pt. 4: *p* = 0.0034, Pt. 5: *p* = 0.0056, Pt. 6: *p* < 0.001, Pt. 7: *p* < 0.001, Pt. 10: *p* = 0.0013, Pt. 11: *p* = 0.02; optimal frequency on the basis of PSD: Pt. 4: *p* = 0.0005, Pt. 5: *p* = 0.0056, Pt. 6: *p* < 0.001, Pt. 11: *p* = 0.02).

Furthermore, we analyzed the interregional network connectivity between the epileptogenic region and the surrounding cortical regions of interest. The GCs also exhibited a decreasing trend after stimulation (optimal frequency on the basis of spike counts: Pt. 1: *p* = 0.017, Pt. 3: *p* = 0.03, Pt. 6: *p* = 0.008; optimal frequency on the basis of PSD: Pt. 4: *p* = 0.03, Pt. 6: *p* = 0.008, Pt. 7: *p* = 0.004). The detailed results are presented in Table 1 and Figure 3.

**FIGURE 5:**
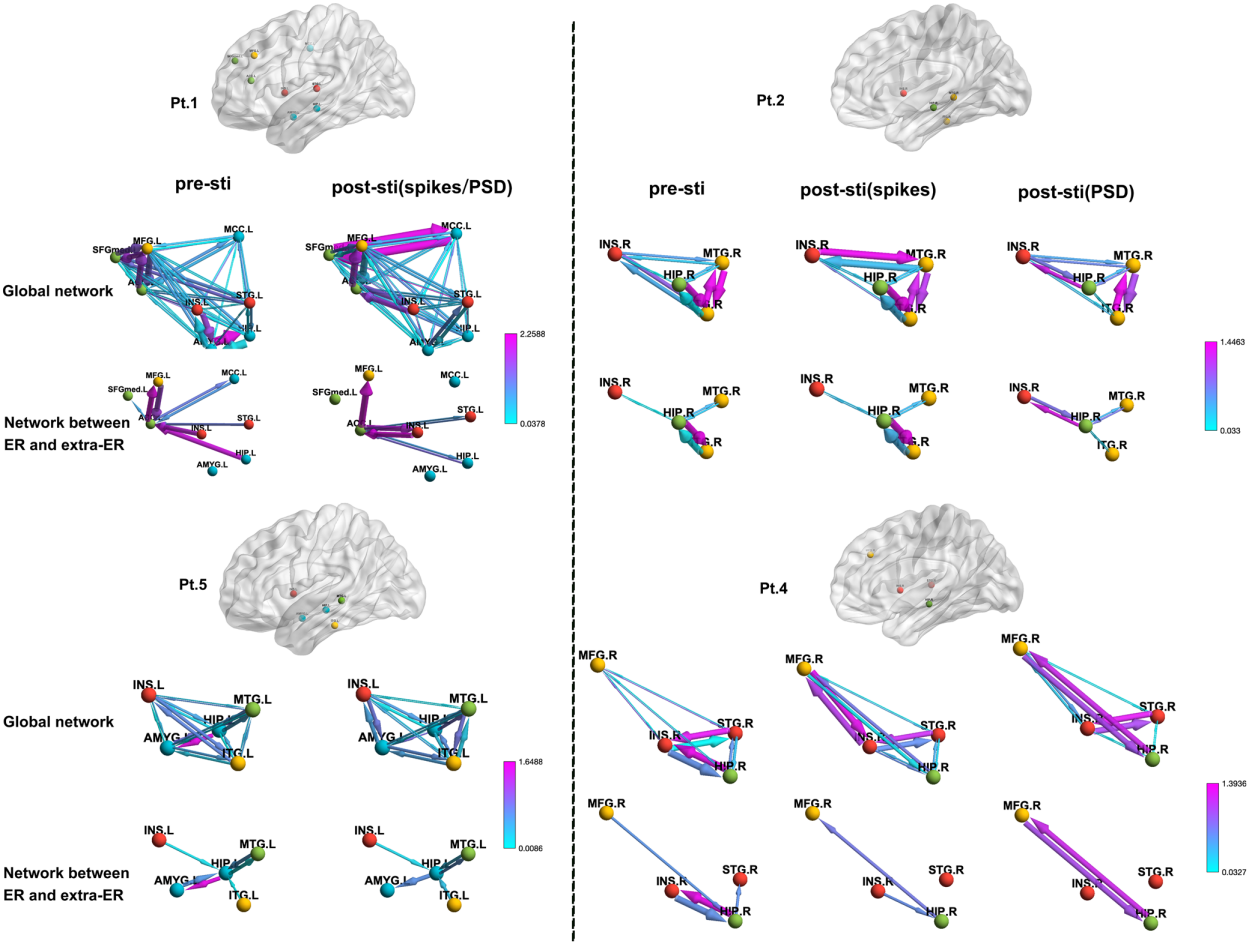
Changes in the Brain Network Before and After Stimulation at the Optimal Frequency. This figure illustrates the changes in the brain network before and after stimulation at the optimal frequency in four representative patients (Pt. 1, Pt. 2, Pt. 4, and Pt. 5) as examples. The epileptogenic region for these patients is located in the ACC (anterior cingulate cortex, Pt. 5) and Hip (hippocampus, Pt. 1, Pt. 2, and Pt. 4). The two cases to the left of the dashed line represent patients (Pt. 1 and Pt. 5) for whom the optimal frequency, which is based on the number of spikes and the PSD as indicators, was consistent, whereas the two cases to the right of the dashed line represent patients (Pt. 2 and Pt. 4) with inconsistent optimal frequencies. The brain surface maps show the projection of selected points for these four patients onto the cortical surface. Below each brain map, changes in network connectivity are presented for the regions of interest (ROIs) and between the epileptogenic region and extra focal cortical regions before and after stimulation.

As illustrative examples, four patients were selected to demonstrate changes in network connectivity within the ROIs and between the epileptogenic region and the surrounding regions before and after stimulation at the optimal frequency. These results are visualized in Figure 5.

**FIGURE 6:**
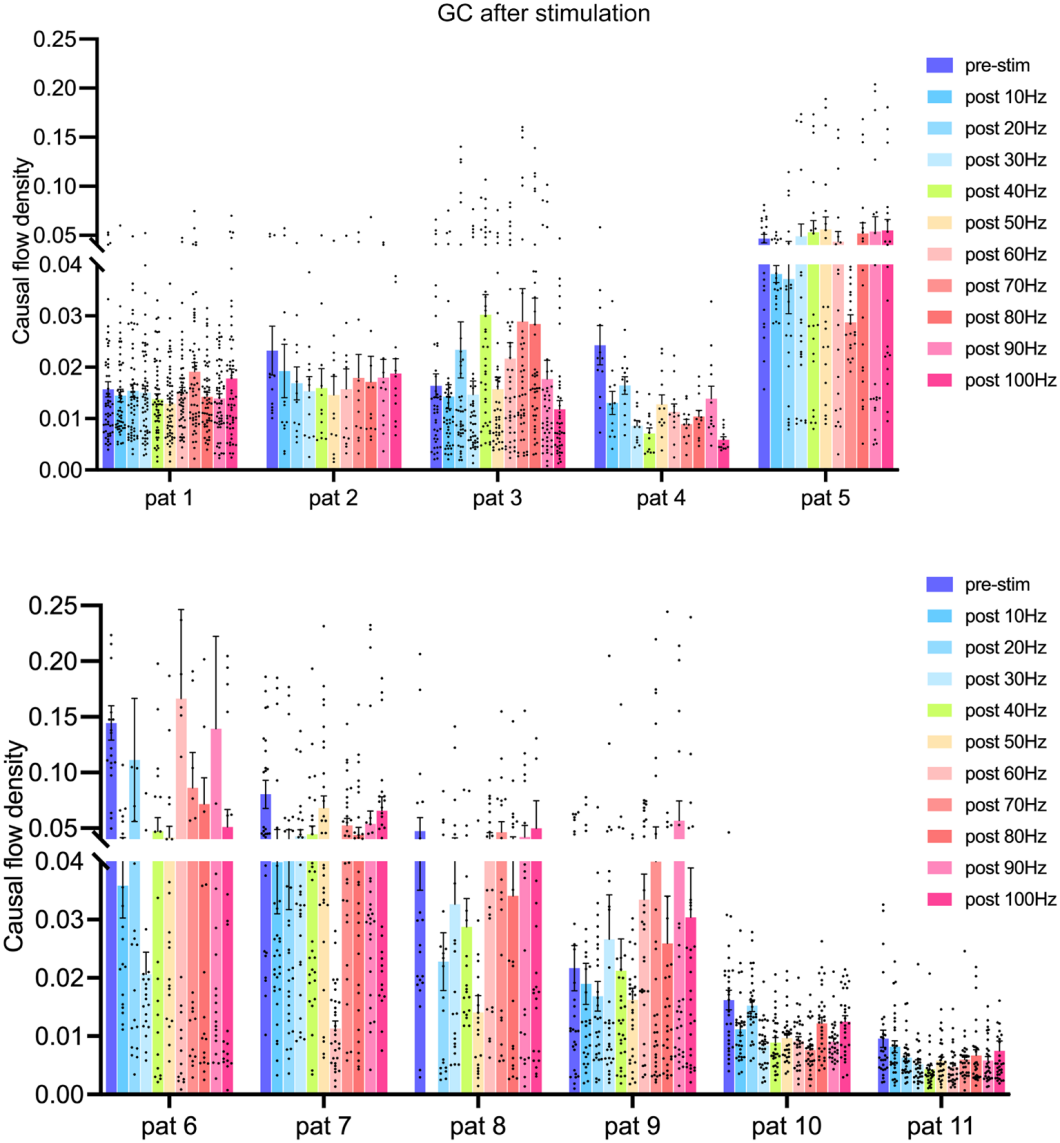
Causal flow changes between regions of interest before and after stimulation at each frequency in all patients. This figure depicts the changes in the brain network within each patient's regions of interest (frontal, temporal, and insular lobes), comparing the brain network within each ROI with that following stimulation at various frequencies (10–100 Hz).

We performed statistical analyses on the network changes in the regions of interest following stimulation at various frequencies across all patients. Although the changes varied among individuals, most patients showed inhibitory effects on overall network connectivity at two or more frequencies within the 10–70 Hz range, as shown in Figure 6.

## 4 Discussion

Currently, the frequencies for ANT‐DBS in the treatment of drug‐resistant epilepsy are often selected on the basis of those used in DBS for movement disorders (90–450 μs pulse width, 1–5 V voltage, 130–150 Hz frequency) [12, 31, 32]. However, whether this fixed high‐frequency stimulation regimen is equally effective in all epilepsy patients requires validation. This study confirmed that individualized selection of stimulation frequencies is likely to significantly enhance the anticonvulsant effects of DBS.

### 4.1 Stimulation in the 10–70 Hz Range Reduces Epileptic Activity

In previous studies, researchers identified spikes as biological markers of epileptogenic tissue, with PSD serving as an indicator of local excitability in epileptogenic foci [33, 34, 35, 36, 37, 38]. Herein, the frequency band showing the most significant reduction in the number of spikes and the PSD within the epileptogenic region varied between patients, but the optimal range was concentrated between 10 and 70 Hz. When a frequency of 10–70 Hz was used to stimulate the ANT, abnormal electrical activity in the epileptogenic foci of patients with frontal or temporal lobe epilepsy was effectively suppressed. This finding has important clinical implications, suggesting that for patients with epileptogenic foci in the frontal or temporal lobes, ANT‐DBS at a frequency of 10–70 Hz could be considered an alternative to conventional stimulation at a frequency of 130–150 Hz, especially when the latter proves ineffective, to maximize the clinical efficacy of ANT‐DBS. Although clinical reports on the use of low‐frequency ANT‐DBS are sparse, they provide preliminary evidence supporting our hypothesis [39, 40, 41].

### 4.2 Stimulation Modulates Connectivity Between Brain Regions

Both resection surgery and neuromodulation therapies for epilepsy aim to disrupt the epileptic network to reduce the occurrence of seizures [42, 43, 44]. Increasing evidence shows that ANT‐DBS not only suppresses local epileptogenic foci but also modulates associated brain networks, gradually restoring them to a more normal state. Previous studies revealed that high‐frequency ANT‐DBS has a neuroregulatory effect on brain networks in drug‐resistant epilepsy patients [10, 45]. In this study, we performed a GC analysis to examine the direct modulatory effects of ANT electrical stimulation at 10–70 Hz on the brain network. In most patients, ANT stimulation at specific frequencies led to a significant reduction in network connectivity between the frontal, temporal, and insular lobes. We also analyzed the changes in GC flow density between epileptogenic regions and surrounding cortical regions, revealing a regulatory influence. These findings suggested that ANT stimulation inhibits local epileptiform activity in the focus region, reduces connectivity between the focus region and other cortical regions, and has significant modulatory effects on network connectivity between different brain regions.

The ANT is extensively connected to the medial temporal lobe as well as the lateral and medial frontal lobes, making it an effective target for modulating epilepsy associated with abnormalities in these regions [46, 47, 48]. In this study, most patients had temporal or frontal lobe epilepsy, further confirming that stimulating the ANT at a frequency of 10–70 Hz not only inhibited local epileptogenic activity but also modulated brain network connectivity, both of which often occur simultaneously. However, in the exceptional case of Pt. 8, stimulating the ANT successfully suppressed local epileptogenic activity, but no significant modulation of the brain network was observed. Imaging and other surgical assessments revealed that this patient's epileptogenic region was extensive, suggesting that network modulation may not be evident in the short term. Long‐term stimulation may be required to induce changes in network connectivity.

### 4.3 Individualized Frequency in the Treatment Protocol

This study confirms that the modulatory effects of ANT stimulation depend on the stimulation frequency. Electrical stimulation of the ANT at different frequencies results in distinct changes in both the local epileptogenic region and brain network connectivity. Although the optimal stimulation frequency for most patients falls within the 10–70 Hz range, there are still significant individual differences. On the one hand, this underscores the need to consider not only electrode contact placement and stimulation current but also the selection of the optimal frequency during the programming phase of ANT‐DBS. On the other hand, frequency‐dependent individualized electrophysiological responses may help explain some adverse reactions in certain patients. In some studies, researchers suggest that high‐frequency stimulation in the 130–150 Hz range may induce side effects such as mood disorders, insomnia, and irritability [49, 50]. In such cases, switching to a lower‐frequency range (10–70 Hz) is a reasonable alternative. This frequency‐specific efficacy may be particularly relevant for patients with temporal or limbic epilepsy or those whose seizures are sustained by hypersynchronous network activity. Preclinical studies have shown that low‐frequency stimulation (LFS, typically 1–10 Hz) of the ANT can reduce seizure frequency, increase seizure thresholds, and modulate pathological rhythms in hippocampal and thalamocortical circuits [51, 52]. Moreover, recent clinical observations suggest that lower frequencies (e.g., 2–7 Hz) may preserve or even enhance cognitive functions while providing seizure control in certain patients [53]. These findings underscore the need for individualized DBS programming strategies based on seizure networks and patient profiles. Future research with larger cohorts will be critical for identifying the epilepsy subtypes and network configurations that are most responsive to low‐frequency ANT‐DBS. Therefore, during long‐term postsurgical adjustments, identifying the optimal stimulation frequency on the basis of patient symptom feedback not only presents a higher level of complexity but also offers greater flexibility for enhancing the clinical efficacy of ANT‐DBS and minimizing adverse effects.

## 5 Limitations

First, only a small number of patients were included in this study; thus, a larger sample is needed in future research for validation. Second, individualized electrode implantation on the basis of the localization of the epileptogenic zone led to intersubject variability, complicating the identification of consistent brain network patterns. Third, owing to some patients' discomfort at stimulation frequencies above 110 Hz and the aim of this study to explore alternative effective frequencies, systematic testing at higher frequencies was not conducted. Fourth, although a 60‐s interval was used between stimulation trials, the possibility of cumulative stimulation effects cannot be entirely excluded. Finally, this study focused on the immediate effects of short‐term ANT stimulation, which may not fully capture the long‐term therapeutic impact of ANT‐DBS. Long‐term DBS is believed to involve slower neuroplastic processes, such as synaptic remodeling and glial modulation, that are not engaged by acute protocols. Thus, while the observed reductions in spike counts or PSD may reflect early network responses, they should be interpreted as transient effects rather than indicative of long‐term efficacy. Additional longitudinal studies are warranted to clarify whether such acute biomarkers can reliably predict chronic treatment outcomes.

## 6 Conclusions

Our study demonstrated that ANT stimulation significantly inhibits epileptogenic regions and modulates the large‐scale brain network. These effects exhibit notable interindividual variability and are frequency dependent. For epilepsy patients who show a suboptimal response to high‐frequency ANT‐DBS, the use of lower frequencies (10–70 Hz) may improve therapeutic outcomes.

## Author Contributions

Ying Gao and Hao Yan helped collect the stimulation data, draft the manuscript, and prepare the figures. Xueyuan Wang, Liang Qiao, Wei Shu, and Duanyu Ni implanted the SEEG electrodes. Ying Gao and Guiliang Hao analyzed the data. Tao Yu helped conceptualize and design the study and optimize the figures. Guoguang Zhao and Liankun Ren optimized the research design and improved the manuscript.

## Ethics Statement

This study was approved by the Medical Ethics Committee of Xuanwu Hospital, Capital Medical University (LYS2018041).

## Consent

Informed consent was obtained from the individual participants included in the study.

## Conflicts of Interest

The authors declare no conflicts of interest.

## Data Availability

The data that support the findings of this study are available upon request from the corresponding author. The data are not publicly available due to privacy and ethical restrictions.
